# HIV risks and needs related to the Sustainable Development Goals among female sex workers who were commercially sexually exploited as children in Lesotho

**DOI:** 10.1002/jia2.25042

**Published:** 2018-02-27

**Authors:** Ashley Grosso, Shianne Busch, Tampose Mothopeng, Stephanie Sweitzer, John Nkonyana, Nkomile Mpooa, Noah Taruberekera, Stefan Baral

**Affiliations:** ^1^ Public Health Solutions Research and Evaluation Unit New York NY USA; ^2^ Department of Epidemiology Johns Hopkins Bloomberg School of Public Health Center for Public Health and Human Rights Baltimore MD USA; ^3^ Matrix Support Group Maseru Lesotho; ^4^ Ministry of Health Maseru Lesotho; ^5^ Care for Basotho Maseru Lesotho; ^6^ Care‐Lesotho Maseru Lesotho; ^7^ Population Services International Johannesburg South Africa

**Keywords:** AIDS, sex trafficking, human trafficking, sexual violence, physical violence, adolescents

## Abstract

**Introduction:**

Sustainable Development Goals (SDGs) about gender equality; decent work; and peace, justice, and strong institutions include a focus on eradicating trafficking and sexual exploitation of and violence against women and children. In Lesotho, 86% of women have experienced gender‐based violence. In addition, overall HIV prevalence is among the highest globally, and higher among adolescent girls than boys. Moreover, nearly three quarters of female sex workers (FSW) are estimated to be living with HIV in Lesotho. In this context, sexually exploited children may be particularly vulnerable to violence and HIV acquisition risks. This study's objective is to examine the prevalence and correlates of experiencing sexual exploitation as a child among FSW in Lesotho.

**Methods:**

FSW (≥18 years) recruited through respondent‐driven sampling in Maseru and Maputsoe from February to September 2014 completed HIV and syphilis testing and an interviewer‐administered survey, including a question about the age at which they started providing sex for money. This study examined correlates of experiencing sexual exploitation as a child (<18 years) through multivariable logistic regression analyses for each city, controlling for current age.

**Results:**

Across both cities, 20.0% (142/710) of participants were sexually exploited as children. Among them, 65.5% (93/142) tested positive for HIV and 31.0% (44/142) for syphilis, which was similar to those who started selling sex as adults, after adjusting for current age. Participants who experienced child sexual exploitation were more likely to have been forced to have sex before age 18 than those who started selling sex as adults (Maseru‐adjusted odds ratio (aOR): 3.52, 95% Confidence Interval (CI): 1.61 to 7.66, *p* = 0.002; Maputsoe‐aOR: 4.39, 95% CI: 1.22 to 15.75, *p *=* *0.023). In Maseru, participants who were sexually exploited as children were more likely to avoid carrying condoms to prevent trouble with police (aOR: 3.18, 95% CI: 1.50 to 6.75, *p *=* *0.003).

**Conclusions:**

Risk determinants for HIV and violence among sexually exploited children can be studied retrospectively through research with adult FSW. Further research working directly with sexually exploited children will improve understanding of their needs. Preventing commercial sexual exploitation of children and addressing the social and healthcare needs of those who are exploited are necessary to fully achieve SDGs 5, 8 and 16 and an AIDS‐Free Generation.

## 1 Introduction

Much of the research on the sexual exploitation of children has been conducted in Asia and the Americas, 1 but studies have increasingly explored this topic in sub‐Saharan Africa 2. For example, studies with adult female sex workers (FSW) in Namibia 3 and Ghana 4 have included questions about the age at which the participant started selling sex, with some women reporting experiencing this before age 18. The sexual exploitation of children in the region has been shown to be associated with socio‐demographic factors, violence, positive and negative interactions with police, and risks for HIV and other sexually transmitted infections.

The death of one or both parents has been reported as a reason for selling sex before the age of 18 in qualitative research in Tanzania and Nigeria 5, 6. Limited education may be the cause of or consequence of experiencing sexual exploitation as a child. In a study in West Africa, the majority of sexually exploited children in Niger had completed primary school or less, and in Benin the majority were uneducated or had dropped out of school 7. Leaving school and inability to afford school fees were also common themes in interviews with women who were sexually exploited as children in Zimbabwe 8 and Ethiopia 9.

Sexually exploited children are also at risk for experiencing physical and sexual violence. In a multi‐country study, compared to FSW who started selling sex as adults, FSW who were sexually exploited as children were more likely to report they had been beaten up in Swaziland and tortured in the Gambia 10. In Uganda, youth who were sexually exploited had five times higher odds of experiencing any rape than youth who were not exploited 11.

Despite being at risk for experiencing violence, sexually exploited children may be unwilling or unable to report these experiences of violence to the police because they may be arrested for selling sex; however many countries in the region have supportive policies, such as in Cameroon where children do not have to be accompanied by a parent or guardian to press charges 12.

Sexually exploited children are often at elevated risk for sexually transmitted infection (STI) and HIV acquisition, particularly in sub‐Saharan Africa due to the high HIV prevalence overall. In Cote d'Ivoire, FSW who were commercially sexually exploited as children were more likely to test positive for HIV than those were not 13. In Mozambique, a higher percentage of sexually exploited youth had recent STI symptoms than adult FSW 14. One reason for this elevated risk may be challenges in using condoms. In Kenya, FSW who were sexually exploited as children had lower condom use self‐efficacy and were less likely to use condoms consistently with clients 15. In Burkina Faso, reporting that clients removed condoms or paid more not to use condoms was more common among FSW who were sexually exploited as children than FSW who were not 16.

Ending sexual exploitation and human trafficking is included in the targets of three Sustainable Development Goals (SDGs) 17: Goal 5, Gender Equality; Goal 8, Decent Work; and Goal 16, Peace, Justice and Strong Institutions. Several SDG indicators are relevant to sexually exploited children and their risks and vulnerabilities. These include the proportion of victims of human trafficking by age and form of exploitation, rape before age 18, intimate partner violence, sexual violence by other types of perpetrators, and reporting violence to the authorities. Table 1 provides more details about these goals, targets and indicators.

**Table 1:** Definitions and Sustainable Development Goals related to trafficking and sexual exploitation and relevant study variables

**Human trafficking** includes using force, coercion or payments to a control another person for exploitation, including prostitution and sexual exploitation 18. Exploitation of a child under 18 years old is considered trafficking even if it does not involve force or coercion.			
**Child sex trafficking** is sometimes defined as recruiting or transporting a child for the purposes of commercial sex, but is also used more generally to refer to the sale of sex with a child under the age of 18 for remuneration to the child or a third person 19.			
**The sexual exploitation of children**, which is sometimes equated with child sex trafficking, includes sale of sex with a child and can also encompass child pornography, child sex tourism, and child marriages 20. In this paper, the term “sexual exploitation of children” is used to refer only to the sale of sex for money with a child under 18 years old 21.			
Goals	Targets	Indicators	Related Survey Questions
5. Achieve gender equality and empower all women and girls	5.2. Eliminate all forms of violence against all women and girls in the public and private spheres, including trafficking and sexual and other types of exploitation	5.2.1. Proportion of ever‐partnered women and girls aged 15 years and older subjected to physical, sexual or psychological violence by a current or former intimate partner in the previous 12 months, by form of violence and by age	“Has someone ever physically hurt you (pushed, shoved, slapped, hit, kicked, choked, or otherwise physically hurt you)?” “Who was the person who physically hurt you? I will read you the following options, and please tell me for each one whether this type of person ever beat you up or physically hurt you.” Husband, boyfriend or any current or past non‐paying sexual partner
		5.2.2. Proportion of women and girls aged 15 years and older subjected to sexual violence by persons other than an intimate partner in the previous 12 months, by age and place of occurrence	“Has someone ever forced you to have sex when you did not want to? (By forced I mean physically forced, coerced to have sex or penetrated with an object, when you did not want to).” “When was the last time someone forced you to have sex?”
8. Promote sustained, inclusive and sustainable economic growth, full and productive employment and decent work for all	8.7. Take immediate and effective measures to eradicate forced labour, end modern slavery and human trafficking and secure the prohibition and elimination of the worst forms of child labour, including recruitment and use of child soldiers, and by 2025 end child labour in all its forms	8.7.1. Proportion and number of children aged 5 to 17 years engaged in child labour, by sex and age	“Approximately how old were you the first time you provided sexual acts in exchange for money?”
16. Promote peaceful and inclusive societies for sustainable development, provide access to justice for all and build effective, accountable and inclusive institutions at all levels	16.1. Significantly reduce all forms of violence and related death rates everywhere	16.1.3 Proportion of population subjected to physical, psychological or sexual violence in the previous 12 months	“Has someone ever physically hurt you (pushed, shoved, slapped, hit, kicked, choked, or otherwise physically hurt you)?” “Approximately how many times has someone physically hurt you in any way in the past 12 months?” “Has someone ever forced you to have sex when you did not want to? (By forced I mean physically forced, coerced to have sex or penetrated with an object, when you did not want to).” “When was the last time someone forced you to have sex?”
	16.2. End abuse, exploitation, trafficking and all forms of violence against and torture of children	16.2.2. Number of victims of human trafficking per 100,000 population, by sex, age and form of exploitation	“What were the reasons you started selling sex? I will read you several possible reasons. For each, please tell me if it was a reason you started selling sex.” I was forced against my will (By forced, I mean physically forced or coerced into selling sex) Someone talked me into it or pressured me (defined as social pressure. For example, a participant was considering it and someone else heavily encouraged or pushed her into selling sex). “Why do you currently sell sex?” I am being coerced or forced Someone is pressuring me to continue selling sex
		16.2.3. Proportion of young women and men aged 18 to 29 years who experienced sexual violence by age 18	“Has someone ever forced you to have sex when you did not want to? (By forced I mean physically forced, coerced to have sex or penetrated with an object, when you did not want to).” “Approximately how old were you the first time someone forced you to have sex? (age in years)”
	16.3. Promote the rule of law at the national and international levels and ensure equal access to justice for all	16.3.1. Proportion of victims of violence in the previous 12 months who reported their victimization to competent authorities or other officially recognized conflict resolution mechanisms	“After someone forced you to have sex, who did you tell about this experience? I will read you the following options, and please tell me for each one whether you told this person about the experience(s).” Uniformed officer (police, military, security officer) “How would you describe your relationship, in general, with police?” (bad, neutral or good) “Have you ever avoided carrying condoms because you were afraid that they might get you in trouble with the police?”

The majority of new HIV infections among young people aged 15 to 24 occur in sub‐Saharan Africa 22. In Lesotho, overall HIV prevalence is among the highest globally 23, and four times higher among adolescent girls than boys 24. Moreover, nearly three quarters of FSW are estimated to be living with HIV in Lesotho 25. Concurrent with this high HIV prevalence is widespread violence against women; 86% of women have experienced gender‐based violence 26, and studies have called for more rigorous evidence on abuse and the exploitation of children. In this context, sexually exploited children may be particularly vulnerable to gender‐based violence and HIV acquisition risks. Given the difficulties of obtaining parental consent for research with sexually exploited children, some studies have asked adult FSW about the age at which they started exchanging sex for money 27, 28. The purpose of this study is to examine the prevalence of experiencing commercial sexual exploitation as a child among adult FSW in two cities in Lesotho and its associations with variables related to the SDG indicators.

## 2 Methods

### 2.1 Sampling

FSW aged 18 years and older were recruited through respondent‐driven sampling (RDS) in Maseru and Maputsoe from February to September 2014 to participate in a survey and biological testing to estimate the prevalence and correlates of HIV infection. The study team chose RDS, a chain‐referral peer‐driven sampling method, because it can generate representative data for hidden populations 29, 30. Eligibility criteria included being assigned female sex at birth, selling sex within the past six months as a principal source of revenue, providing verbal informed consent in Sesotho or English, having a valid recruitment coupon, and living in Lesotho for at least the past three months. Seven and 12 “seeds” (initial participants) in Maseru and Maputsoe respectively, were each given up to three coupons to recruit other FSW into the study, who were then given up to three coupons after participation until the study ended. The sample size was calculated using the assumption that HIV prevalence among FSW in Lesotho would be similar to levels observed in Swaziland, where the HIV prevalence was 61.0% (95% CI: 52.1 to 69.0) among FSW 31. The formula used for this study to calculate the sample size was:

$$n=\text{deff.}\frac{P_{A}\left(1-P_{A}\right)}{{\left(\text{se}({\hat{P}}_{A})\right)}^{2}}$$

where n = sample size, deff = design effect and P = assumed prevalence 32. Assuming HIV prevalence in Lesotho FSW could have been as low as 52%, the lower limit of the 95% confidence interval (CI) of the Swaziland FSW HIV prevalence estimate, design effect of 2, and a standard error no greater than 0.035, it was estimated that a sample size of 408, rounded off to 410, FSW per site was needed. Homophily, the tendency for participants to recruit others like them, was low (under ±0.25). Convergence was achieved for the variable on child sexual exploitation, defined using the criteria that the required number of recruitment waves estimated to reach equilibrium was smaller than the number of waves in the RDS sample.

### 2.2 Survey administration and biological testing

The study took place in Maseru in rooms leased at a sexual health clinic, while in Maputsoe space was rented at a hotel connected to a bar where FSW work. Whole blood samples were drawn from participants by trained nurse counsellors for HIV testing using Determine Rapid Test (Alere Waltham, Massachusetts, USA) and syphilis testing using Unigold Rapid Test (Trinity, Ireland), consistent with national guidelines. Participants who tested positive for active syphilis were offered free treatment. Participants who tested positive for HIV were referred to treatment and care services during post‐test counselling. Prior to biological testing, participants completed an interviewer‐administered survey in a private room. Participants who completed the survey and biological testing were given 20 LSL (approximately 2 USD) as reimbursement for their time, 26 LSL (approximately 2.60 USD) as reimbursement for transport, male and/or female condoms, and HIV information materials. Participants also received 20 LSL (about 2 USD) for each eligible participant they recruited and another 26 LSL for transport if they returned to collect their recruiter reimbursement and complete a post‐recruitment questionnaire. The Population Services International Research Ethics Board and the National Health Research Ethics Committee of Lesotho approved the study.

The survey was adapted from studies with FSW across sub‐Saharan Africa 16, 33, 34, 35, 36, 37 and pre‐tested with FSW community members to ensure that questions were understood in English and Sesotho. Topics included socio‐demographic characteristics, human rights violations, sexual behaviour, health service access, mental health, social capital, and reproductive health. Survey data were double‐entered using EpiData (Odense, Denmark).

### 2.3 Analytical methods

To measure child sexual exploitation, the dependent variable was dichotomized such that those who reported that they exchanged sex for money at any age less than 18 years old were coded as 1 and those who answered any age greater than or equal to 18 years old were coded as 0.

Potential independent variables were considered based on associations with child sexual exploitation in prior studies and guided by the five levels of the modified social ecological model for characterizing HIV risk among key populations 38: individual, network, community, policy, and stage of the HIV epidemic. The independent variables were also chosen based on their relevance to the SDG indicators. Additional variables in the datasets that were not significantly related to sexual exploitation as a child in bivariate models or in models adjusting for current age for both cities were considered not to be potential confounders and were excluded as beyond the scope of this paper. For the multivariable models, variables were selected that were applicable to all FSW in the study (rather than, for example, only those who had non‐paying partners).

The survey questions from which independent variables related to the SDGs were derived are included in Table 1. In addition, demographic variables included orphanhood (whether at least one of the participant's parents died before she was 18 years old) and education (completed primary school or less). As a structural determinant of HIV risk, a variable indicating whether the participant usually buys all her condoms, gets them for free, both, or neither was included. HIV and syphilis prevalence among participants who were sexually exploited as children and those who were not is also reported.

Correlates of experiencing sexual exploitation as a child were analysed using Stata/SE 14.1 (College Station, TX, USA). Each city's dataset was analysed separately because participants were recruited through separate networks, and the distribution of responses varied substantially by city for the variables of interest. Because missing data were less than six percent, listwise deletion was used. Missing data are indicated in the tables, with denominators adjusted. Participants with missing data on whether they were sexually exploited as children were excluded from the analyses. Factors significantly associated with experiencing child sexual exploitation in bivariate models were entered into multivariable logistic regression models. Due to collinearity, the only physical violence variable included was ever experiencing violence. Because of its relevance to experiencing commercial sexual exploitation before age 18, and due to collinearity, being forced to have sex before age 18 was the only sexual violence variable included. After adjusting for age, HIV status and being orphaned before age 18 were no longer statistically significantly related to experiencing sexual exploitation as a child and were excluded from further multivariable models. The Akaike Information Criterion was used to select the most parsimonious models. All multivariable analyses controlled for age at the time of the survey. Analyses were not adjusted for respondent driven sampling weighting. The variance inflation factor (VIF) was calculated to test multicollinearity. The final model had a mean VIF of 1.37 in Maseru and 2.05 in Maputsoe. The overall multivariable models were significant at the 0.05 level according to the likelihood ratio chi‐square statistic. The model predicted 81.8% of the responses correctly in Maseru and 83.8% in Maputsoe. The Pseudo R^2^ was 0.20 in Maseru and 0.19 in Maputsoe.

## 3 Results

### 3.1 Socio‐demographics

Characteristics of participants in Maseru, Maputsoe, and the two cities combined (n = 744) are reported in Table 2. As shown in Tables 3 and 4 respectively, the mean age of participants who were sexually exploited as children was about 22 in Maseru and about 24 in Maputsoe at the time of the survey. This was younger than the mean age of participants who were not exploited (Maseru‐mean age: 21.9 vs. 25.7, odds ratio (OR): 0.81, 95% CI: 0.75 to 0.87, adjusted odds ratio (aOR): 0.82, 95% CI 0.76 to 0.89; Maputsoe‐mean age: 23.9 vs. 30.0, OR: 0.87, 95% CI: 0.82 to 0.92, aOR: 0.87, 95% CI: 0.82 to 0.93). Most participants completed primary school or less. Over three quarters of participants who were sexually exploited as children in Maseru and over 60% in Maputsoe reported at least one parent had died during the participant's childhood. In Maseru, this was more likely than among participants who were not exploited in the bivariate analysis (Table 3, 77.7% vs. 59.5%, OR: 2.37, 95% CI: 1.35 to 4.15).

**Table 2:** Characteristics of female sex workers recruited through respondent‐driven sampling in Lesotho, 2014

	Maseru	Maputsoe	Combined
Socio‐demographics			
Age at time of survey (mean)	24.8	29.1	26.8
Education			
Completed primary school or less	56.7% (232/409)	63.1% (210/333)	59.6% (442/742)
Orphaned before age 18			
At least one parent died	64.6% (255/395)	52.0% (169/325)	58.9% (424/720)
Entry into and reasons for selling sex			
Started selling sex <18	22.5% (89/395)	16.8% (53/315)	20.0% (142/710)
Was forced or coerced to start selling sex	0.5% (2/410)	0.0% (0/333)	0.3% (2/743)
Was talked into or pressured to start selling sex	16.8% (69/410)	9.3% (31/333)	13.5% (100/743)
Currently forced or coerced to sell sex	0.2% (1/410)	0.0% (0/333)	0.1% (1/743)
Currently talked into or pressured to sell sex	0.2% (1/410)	0.0% (0/333)	0.1% (1/743)
Experiences of violence			
Experienced physical violence ever	58.5% (240/410)	22.5% (75/333)	42.4% (315/743)
Experienced physical violence from an intimate partner ever	18.6% (76/409)	9.0% (30/333)	14.3% (106/742)
Experienced physical violence from a uniformed officer (police, military, security officer) ever	19.1% (78/409)	0.9% (3/333)	10.9% (81/742)
Experienced physical violence in the past 12 months	53.2% (218/410)	18.3% (61/333)	37.6% (279/743)
Was ever forced to have sex	42.0% (172/410)	15.1% (50/332)	29.9% (222/742)
Was forced to have sex before age 18	10.2% (42/410)	4.8% (16/332)	7.8% (58/742)
Was ever forced to have sex by an intimate partner	7.1% (29/410)	3.3% (11/332)	5.4% (40/742)
Was ever forced to have sex by a uniformed officer	7.3% (30/410)	0.3% (1/332)	4.2% (31/742)
Ever told uniformed officer about being raped	12.2% (21/172)	20.0% (10/50)	14.0% (31/222)
Relationship with authorities			
Relationship with police			
Neutral	18.8% (77/409)	16.8% (56/333)	17.9% (133/742)
Bad	48.2% (197/409)	5.7% (19/333)	29.1% (216/742)
Good	33.0% (135/409)	77.5% (258/333)	53.0% (393/742)
Ever avoided carrying condoms out of fear of trouble with police	12.0% (49/410)	1.5% (5/333)	7.3% (54/743)
Sexually transmitted infections and access to prevention			
Condom acquisition			
Buy all	9.8% (40/410)	3.9% (13/333)	7.1% (53/743)
Get all for free	73.9% (303/410)	87.1% (290/333)	79.8% (593/743)
Buy and get for free	15.6% (64/410)	8.1% (27/333)	12.2% (91/743)
Neither	0.7% (3/410)	0.9% (3/333)	0.8% (6/743)
Laboratory results			
Living with HIV	73.1% (299/409)	70.4% (235/334)	71.9% (534/743)
Active syphilis	27.9% (114/409)	26.4% (88/334)	27.2% (202/743)

**Table 3:** Prevalence and correlates of experiencing sexual exploitation as a child among female sex workers in Maseru, Lesotho

	Started selling sex <18 (22.5%, 89/395)	Started selling sex 18+ (77.5%, 306/395)	OR (95% CI)	*p*	aOR^a^ (95% CI)	*p*
Socio‐demographics						
Age at time of survey (mean)	21.9	25.7	**0.81 (0.75 to 0.87)**	**<0.001**	**0.81 (0.75 to 0.88)**	**<0.001**
Education						
Completed primary school or less	56.2% (50/89)	56.4% (172/305)	0.99 (0.62 to 1.60)	0.971	‐	‐
Orphaned before age 18						
At least one parent died	77.7% (66/85)	59.5% (176/296)	**2.37 (1.35 to 4.15)**	**0.003**	‐	‐
Entry into and reasons for selling sex						
Was forced or coerced to start selling sex	0.0% (0/89)	0.7% (2/306)	‐	‐	‐	‐
Was talked into or pressured to start selling sex	28.1% (25/89)	12.1% (37/306)	**2.84 (1.60 to 5.05)**	**<0.001**	**3.11 (1.60 to 6.04)**	**0.001**
Currently forced or coerced to sell sex	0.0% (0/89)	0.3% (1/306)	‐	‐	‐	‐
Currently talked into or pressured to sell sex	0.0% (0/89)	0.3% (1/306)	‐	‐	‐	‐
Experiences of violence						
Experienced physical violence ever	60.7% (54/89)	58.8% (180/306)	1.08 (0.67 to 1.75)	0.755	‐	‐
Experienced physical violence from an intimate partner ever	19.1% (17/89)	18.4% (56/305)	1.05 (0.57 to 1.92)	0.874	‐	‐
Experienced physical violence from a uniformed officer (police, military, security officer) ever	18.0% (16/89)	20.0% (61/305)	0.88 (0.48 to 1.61)	0.672	‐	‐
Experienced physical violence in the past 12 months	53.9% (48/89)	53.9% (165/306)	1.00 (0.62 to 1.61)	0.999	‐	‐
Was ever forced to have sex	56.2% (50/89)	38.2% (117/306)	**2.07 (1.28 to 3.34)**	**0.003**	‐	‐
Was forced to have sex before age 18	23.6% (21/89)	5.9% (18/306)	**4.94 (2.50 to 9.78)**	**<0.001**	**3.52 (1.61 to 7.66)**	**0.002**
Was ever forced to have sex by an intimate partner	9.0% (8/89)	6.5% (20/306)	1.41 (0.60 to 3.33)	0.429	‐	‐
Was ever forced to have sex by a uniformed officer	6.7% (6/89)	7.8% (24/306)	0.85 (0.34 to 2.15)	0.730	‐	‐
Ever told uniformed officer about being raped	12.0% (6/50)	12.8% (15/116)	0.93 (0.34 to 2.55)	0.884	‐	‐
Relationship with authorities						
Relationship with police						
Neutral	19.1% (17/89)	18.4% (56/305)	Ref.	Ref.	Ref.	Ref.
Bad	52.8% (47/89)	47.5% (145/305)	1.07 (0.57 to 2.01)	0.840	‐	‐
Good	28.1% (25/89)	34.1% (104/305)	0.79 (0.39 to 1.59)	0.511	‐	‐
Ever avoided carrying condoms out of fear of trouble with police	20.2% (18/89)	10.1% (31/306)	**2.25 (1.19 to 4.25)**	**0.013**	**3.18 (1.50 to 6.75)**	**0.003**
Sexually transmitted infections and access to prevention						
Condom acquisition						
Buy all	20.2% (18/89)	7.2% (22/306)	Ref.	Ref.	Ref.	Ref.
Get all for free	65.2% (58/89)	76.1% (233/306)	**0.30 (0.15 to 0.60)**	**0.001**	**0.35 (0.16 to 0.75)**	**0.007**
Buy and get for free	13.5% (12/89)	16.0% (49/306)	**0.30 (0.12 to 0.73)**	**0.008**	**0.28 (0.10 to 0.76)**	**0.012**
Neither	1.1% (1/89)	0.7% (2/306)	0.61 (0.05 to 7.30)	0.697	0.50 (0.03 to 7.14)	0.608
Laboratory results						
Living with HIV	61.8% (55/89)	77.1% (235/305)	**0.48 (0.29 to 0.80)**	0.005	‐	‐
Active syphilis	32.6% (29/89)	26.2% (80/305)	1.36 (0.82 to 2.27)	0.239	‐	‐

a Multivariable logistic regression analyses included the following variables: age at the time of the survey, was talked into or pressured to start selling sex, was ever forced to have sex before age 18, ever avoided carrying condoms out of fear of trouble with police, and condom acquisition. aOR, adjusted odds ratio.

The bold values are statistically significant (*p*<0.05).

**Table 4:** Prevalence and correlates of experiencing sexual exploitation as a child among female sex workers in Maputsoe, Lesotho

	Started selling sex <18 (16.8%, 53/315)	Started selling sex 18+ (83.2%, 262/315)	OR (95% CI)	*p*	aOR^a^ (95% CI)	*p*
Socio‐demographics						
Age at time of survey (mean)	23.9	30.0	**0.87 (0.82 to 0.92)**	**<0.001**	**0.87 (0.82 to 0.93)**	**<0.001**
Education						
Completed primary school or less	62.3% (33/53)	63.7% (167/262)	0.94 (0.51 to 1.73)	0.839	‐	‐
Orphaned before age 18						
At least one parent died	61.5% (32/52)	48.6% (124/255)	1.69 (0.92 to 3.11)	0.092	‐	‐
Entry into and reasons for selling sex						
Was forced or coerced to start selling sex	0.0% (0/53)	0.0% (0/262)	‐	‐	‐	‐
Was talked into or pressured to start selling sex	9.4% (5/53)	9.5% (25/262)	0.99 (0.36 to 2.71)	0.981	‐	‐
Currently forced or coerced to sell sex	0.0% (0/53)	0.0% (0/262)	‐	‐	‐	‐
Currently talked into or pressured to sell sex	0.0% (0/53)	0.0% (0/262)	‐	‐	‐	‐
Experiences of violence						
Experienced physical violence ever	41.5% (22/53)	18.3% (48/262)	**3.16 (1.69 to 5.94)**	**<0.001**	**2.78 (1.39 to 5.56)**	**0.004**
Experienced physical violence from an intimate partner ever	18.9% (10/53)	7.3% (19/262)	**2.97 (1.29 to 6.83)**	**0.010**	‐	‐
Experienced physical violence from a uniformed officer (police, military, security officer) ever	1.9% (1/53)	0.8% (2/262)	2.50 (0.22 to 28.08)	0.458	‐	‐
Experienced physical violence in the past 12 months	32.1% (17/53)	16.0% (42/262)	**2.47 (1.27 to 4.81)**	**0.008**	‐	‐
Was ever forced to have sex	20.8% (11/53)	13.0% (34/261)	1.75 (0.82 to 3.72)	0.147	‐	‐
Was forced to have sex before age 18	11.3% (6/53)	3.1% (8/261)	**4.04 (1.34 to 12.17)**	**0.013**	**4.39 (1.22 to 15.75)**	**0.023**
Was ever forced to have sex by an intimate partner	0.0% (0/53)	3.5% (9/261)	‐	‐	‐	‐
Was ever forced to have sex by a uniformed officer	0.0% (0/53)	0.4% (1/261)	‐	‐	‐	‐
Ever told uniformed officer about being raped	36.4% (4/11)	17.7% (6/34)	2.67 (0.59 to 12.10)	0.204	‐	‐
Relationship with authorities						
Relationship with police						
Neutral	5.7% (3/53)	20.2% (53/262)	Ref.	Ref.	Ref.	Ref.
Bad	9.4% (5/53)	4.6% (12/262)	**7.36 (1.54 to 35.12)**	**0.012**	**9.99 (1.78 to 56.00)**	**0.009**
Good	84.9% (45/53)	75.2% (197/262)	**4.04 (1.21 to 13.50)**	**0.024**	**6.34 (1.64 to 24.49)**	**0.007**
Ever avoided carrying condoms out of fear of trouble with police	1.9% (1/53)	1.5% (4/262)	1.24 (0.14 to 11.32)	0.849	‐	‐
Sexually transmitted infections and access to prevention						
Condom acquisition						
Buy all	3.8% (2/53)	4.2% (11/262)	Ref.	Ref.	Ref.	Ref.
Get all for free	84.9% (45/53)	87.4% (229/262)	1.08 (0.23 to 5.04)	0.921	‐	‐
Buy and get for free	11.3% (6/53)	7.3% (19/262)	1.74 (0.30 to 10.14)	0.540	‐	‐
Neither	0.0% (0/53)	1.2% (3/262)	‐	‐	‐	‐
Laboratory results						
Living with HIV	71.7% (38/53)	69.5% (182/262)	1.11 (0.58 to 2.14)	0.747	‐	‐
Active syphilis	28.3% (15/53)	25.6% (67/262)	1.15 (0.59 to 2.22)	0.680	‐	‐

a Multivariable logistic regression analyses included the following variables: age at time of survey, experienced physical violence ever, was ever forced to have sex before age 18, and relationship with police. aOR, adjusted odds ratio.

The bold values are statistically significant (*p*<0.05).

### 3.2 Entry into and reasons for exchanging sex for money

In Maseru and Maputsoe, 22.5% (89/395) and 16.8% (53/315) of FSW started exchanging sex for money before the age of 18 respectively. None who experienced child sexual exploitation said they were initially or currently forced or coerced into selling sex. None who were exploited were *currently* pressured to sell sex, but 28.1% in Maseru and 9.4% in Maputsoe were talked into or pressured to *start* selling sex. FSW who were sexually exploited as children were more likely to have been talked into or pressured to start selling sex than those who were not exploited (Table 3, 28.1% vs. 12.1%, OR: 2.84, 95% CI: 1.60 to 5.05, aOR: 3.11, 95% CI: 1.60 to 6.04).

### 3.3 Violence

Over half of women who were exploited from Maseru had been physically assaulted ever and in the past 12 months, but fewer women from Maputsoe reported this. About 19% in both cities had ever been physically assaulted by a non‐paying sexual partner. Compared to participants who were not exploited, FSW in Maputsoe who were sexually exploited as children were more likely to have experienced physical violence ever (Table 4, 41.5% vs. 18.3%, OR: 3.16, 95% CI: 1.69 to 5.94, aOR: 2.78, 95% CI: 1.39 to 5.56), in the past 12 months (32.1% vs. 16.0%, OR: 2.47, 95% CI: 1.27 to 4.81), and from an intimate partner (18.9% vs. 7.3%, OR: 2.97, 95% CI: 1.29 to 6.83).

One fifth of participants who were sexually exploited as children in Maputsoe and over half of participants in Maseru had ever been forced to have sex. In Maseru, this was more common than among those who were not exploited (Table 3, 56.2% vs. 37.9%, OR: 2.07, 95% CI: 1.28 to 3.34). In Maseru 23.6% and in Maputsoe 11.3% of those who were exploited were forced to have sex before the age of 18. This was more common among those who were sexually exploited as children than among those who were not (Maseru‐23.6% vs. 5.9%, OR: 4.94, 95% CI: 2.50 to 9.78, aOR: 3.52, 95% CI: 1.61 to 7.66; Maputsoe‐11.3% vs. 3.1%, OR: 4.04, 95% CI: 1.34 to 12.17, aOR: 4.39, 95% CI: 1.22 to 15.75). Nine percent of FSW who were exploited in Maseru and none in Maputsoe reported at least one incident of being forced to have sex by a non‐paying sexual partner in their lifetime. Twelve percent in Maseru and 36.4% in Maputsoe ever reported being forced to have sex by any perpetrator to the police or authorities.

### 3.4 Relationship with authorities

Most participants who were commercially sexually exploited as children in Maseru had a bad relationship with police, while most in Maputsoe had a good relationship with police. In Maputsoe, compared to those who were not exploited they were less likely to have a neutral relationship with police (Table 4, 5.7% vs. 20.2%) compared to a bad (OR: 7.36, 95% CI: 1.54 to 35.12, aOR: 9.99, 95% CI: 1.78 to 56.00) or good relationship (OR: 4.04, 95% CI: 1.21 to 13.50, aOR: 6.34, 95% CI: 1.64 to 24.49). One fifth of participants who were exploited in Maseru and less than 2% in Maputsoe avoided carrying condoms due to potential police conflict. In Maseru this was more likely than among those who were not exploited (Table 3, 20.2% vs. 10.1%, OR: 2.25, 95% CI: 1.19 to 4.25, aOR: 3.18, 95% CI: 1.50 to 6.75).

### 3.5 HIV and STI risks

Over 60% of FSW in Maseru and over 70% in Maputsoe who were exploited tested positive for HIV. In Maseru this was lower than the HIV prevalence among those who were not exploited (Table 3, 61.8% vs. 77.1%, OR: 0.48, 95% CI: 0.29 to 0.80). However this was no longer statistically significant after adjusting for current age. Nearly one third of those exploited in Maseru and over one quarter in Maputsoe had active syphilis. Most participants received all their condoms for free. In Maseru, FSW who were exploited as children were more likely to have bought all their condoms (Table 3, 20.2% vs. 7.2%) rather than getting them all for free (OR: 0.30, 95% CI: 0.15 to 0.60, aOR: 0.35, 95% CI: 0.16 to 0.75) or buying some and getting some for free (OR: 0.30, 95% CI: 0.12 to 0.73, aOR: 0.28, 95% CI: 0.10 to 0.76).

## 4 Discussion

In this study, 20.0% (142/710) of FSW study participants sampled in Maseru and Maputsoe, Lesotho were sexually exploited as children. This is comparable to estimates from Kenya 15, 39, Mozambique 14, and Sudan 40. HIV prevalence was extraordinarily high among this sample of FSW in Lesotho compared to that reported in other settings.

In this study, many FSW who were exploited as children had ever and recently experienced physical and sexual violence from intimate partners and others, which are outcomes that are related to indicators 5.2.1 and 5.2.2. Violence has been shown to be associated with HIV risk 35, 37. Addressing targets of SDG 5 could potentially reduce sexual exploitation of children, violence and adolescent HIV. Given the increased attention to sex trafficking in the current United States presidential administration, concurrent with proposed reductions in funds through the President's Emergency Plan for AIDS Relief, framing requests for resource allocation for reducing sexual exploitation of children as also addressing HIV or vice versa may be a practical strategy in the current funding environment.

Many women in this study, regardless of whether they experienced sexual exploitation as children, had been orphaned and had low education, which may have contributed to their entry into the sex trade. In Maseru, those who were sexually exploited as children were more likely to say they were talked into or pressured to sell sex than those who started as adults. It has been posited that, despite the higher education levels of girls than boys in Lesotho, girls who are orphaned (including those whose parents died of AIDS) may drop out of school in order to financially support their families, including some through selling sex 41. Ending the HIV epidemic, which is part of SDG Target 3.3, may have the additional effect of reducing the number of children orphaned due to AIDS, which may in turn reduce the number of sexually exploited children who are vulnerable to HIV. In this way, HIV programming can contribute to and benefit from the SDG agenda in a bi‐directional way. Addressing the economic and educational needs of orphans and vulnerable children could contribute toward achieving Target 8.7 (eliminating the worst forms of child labour) and reduce the HIV risks associated with selling sex at a young age. Cash transfers and social support have been found to reduce transactional sex among adolescent girls elsewhere in sub‐Saharan Africa and could be considered for preventing the commercial sexual exploitation of girls in Lesotho 42. To mitigate the effects of child sexual exploitation, other programmes in Zimbabwe have provided health education and services bringing together resources from programmes for FSW and programmes for adolescents 43.

The relatively low percentage of participants in this study who reported to police that they had been raped may be related to the finding that bad relationships with police and fear of trouble with police due to carrying condoms disproportionally affect women who started selling sex as minors. However, women who were experienced child sexual exploitation who reported a good relationship with police may have been viewed by officers as “trafficking victims” rather than “sex workers” and thus perceived as more worthy of support 44. Increased protection of these women and decreased enforcement of laws prohibiting selling sex represent an important component of a comprehensive response to decrease significant HIV acquisition and transmission risks observed here and may contribute toward improvements in SDG Indicator 16.3.1.

### 4.1 Limitations

This study's methods have some limitations. This is a secondary analysis, and sample size calculations were based on HIV prevalence rather than child sexual exploitation or other variables in these models. However using the same formula, the sample size was greater than the minimum required (Maseru = 282, Maputsoe = 226) to detect the prevalence of experiencing child sexual exploitation observed here. The data are cross‐sectional, and causal inferences cannot be made. The study data are from 2014, and no follow‐up surveys have been conducted (as is the case with other studies of sexual health in Lesotho, including the Demographic and Health Surveys). However since many of this study's measures were of lifetime experiences (e.g. ever experiencing physical violence), and most participants had been selling sex for three years or more, these results are still potentially relevant in 2017. Self‐reported data may be subject to inaccurate recall and social desirability bias. Offering money for recruitment may have resulted in oversampling of lower income FSW. This may skew the results, particularly related to buying condoms or receiving them for free. Male sex workers were not included, therefore this research does not reflect sexual exploitation experiences among boys 45, 46. The age at which participants acquired HIV is unknown. Participants were not asked whether they would describe their experiences as minors selling sex as exploitation or trafficking 5. The language used to describe this topic is challenging. The term commercially sexually exploited rather than minors who sell sex was chosen for this paper based on feedback from reviewers and international guidelines. The study participants are adults, and their experiences differ from those who were commercially sexually exploited as children and did not continue to sell sex after age 18. Understanding the experiences of sexually exploited children necessitates overcoming restrictions on minors participating in studies without parental consent and using a trauma‐informed research approach to reach this hidden and vulnerable population. Despite these limitations, this study provides evidence of the commercial sexual exploitation of children in an understudied region with high HIV prevalence.

## 5 Conclusions

This study's results indicate that the commercial sexual exploitation of children is prevalent in Maseru and Maputsoe, Lesotho. Experiencing child sexual exploitation in this setting is related to experiencing violence and legal and economic barriers to condom use. Funders of HIV prevention services have given increased attention to understanding specific vulnerabilities among adolescent girls 47. Sexually exploited children are a very vulnerable group whose determinants of risk can be studied retrospectively through research with adult FSW. Further research (where legally and ethically appropriate) with sexually exploited children is needed to overcome limitations of research with adults including inaccurate recall and survival bias. Addressing the issue of commercial sexual exploitation of children is necessary to achieve the targets of the SDGs and an AIDS‐free generation.

## Competing interests

The authors have no competing interests to declare.

## Authors' contributions

SB, TM, JN, NMN, NT and AG collaborated on the design of the study. AG and SB analyzed the data and wrote the paper. All authors provided critical intellectual input into the interpretation of results.
